# The mediating role of transmembrane protein 132D methylation in predicting the occurrence of panic disorder in physical abuse

**DOI:** 10.3389/fpsyt.2022.972522

**Published:** 2022-08-11

**Authors:** Qianmei Yu, Chiyue Wang, Huazheng Xu, Yun Wu, Huachen Ding, Na Liu, Ning Zhang, Chun Wang

**Affiliations:** ^1^School of Psychology, Nanjing Normal University, Nanjing, China; ^2^Nanjing Brain Hospital Affiliated to Nanjing Medical University, Nanjing, China

**Keywords:** panic disorder, childhood trauma, emotional abuse, TMEM132D, gene methylation

## Abstract

**Objective:**

Genome Wide Association study (GWAS) has revealed that the transmembrane protein 132D (TMEM132D) is a gene of sensitive for panic disorder (PD). As the main type of childhood trauma experience, childhood abuse has become a public health issue attracting much attention at home and abroad, and has been proved to be a risk factor for the onset of PD. However, how it affects the occurrence and development of panic disorder has not yet been revealed. We examined the relationship between TMEM132D methylation, childhood abuse and symptoms based on this finding.

**Materials and methods:**

Thirty-two patients with PD and 22 healthy controls (HCs) were recruited after age, gender, and the education level were matched. The DNA methylation levels of CpG sites across the genome were examined with genomic DNA samples (PD, *N* = 32, controls, *N* = 22) extracted from subjects’ elbow venous blood. A mediation model was used to explore the relationship between the methylation degree of different CpG sites and childhood maltreatment and clinical symptoms.

**Results:**

We found that the PD group had significantly lower methylation at CpG1, CpG2, CpG3, CpG4, CpG5, CpG6, CpG7, CpG8, CpG11, CpG14, and CpG18 than did the HCs (*p* < 0.05). The CpG2 (*r* = 0.5953, *p* = 0.0117) site in the priming region of TEME132D gene were positively associated with PDSS score. The CpG2 (*r* = 0.4889, *p* = 0.046) site in the priming region of TEME132D gene were positively associated with physical abuse. Furthermore, path analyses showed that the methylation of CpG2 of TMEM132D played a fully mediating role in the relationship between physical abuse and PD symptom severity (95

**Conclusion:**

Childhood abuse experiences, especially physical abuse, are significantly related to PD. The methylation of CpG2 of TMEM132D was shown to have a fully mediating effect between panic disorder and physical abuse. The interaction between TMEM132D methylation and physical abuse can predict panic disorder.

## Introduction

Panic disorder (PD) is one of anxiety disorders, which is characterized by recurrent unexpected panic attacks and anticipatory anxiety. The first national epidemiological survey of mental disorders in China in 2019 showed that the prevalence of anxiety disorders was higher than other mental disorders, with a weighted annual prevalence rate of 4.98% and a weighted lifetime prevalence rate of 7.57% (1). It is generally believed that PD is a multifactorial disease that is affected by a variety of genetic factors and environmental factors (2). The risk factors affecting the onset of PD are divided into three categories, namely, environment, genetics and physiology.

Epigenetics is one of the biological fields that is considered to play an important role in the etiology of complex diseases (3). Epigenetics refers to potentially heritable and functionally relevant modifications to gene expression and chromatin structure with no changes to genetic sequences (3–6). DNA methylation is one of the major forms of epigenetic modifications (7). Studies have shown that DNA methylation can regulate gene expression, and its mechanism is mainly reflected in the following two ways: methylation directly interferes with the binding site of transcription factors and promoters, and terminates gene transcription or the specific binding of the methylated promoter sequence to the methyl binding protein (MBPs) inhibits the binding of the transcription factor to the target sequence, thereby indirectly inhibiting gene transcription (8). A part of the DNA methylation is reported to be involved in the pathogenesis of psychiatric disorders, including anxiety disorders (9, 10).

Previous studies on DNA methylation in anxiety disorders have focused on candidate genes reported to be related to stress response, neurotransmission and neuroplasticity (11). A recent study of patients with social anxiety disorder (SAD) reported that SAD is associated with increased oxytocin receptor (OXTR) gene high methylation (12). Another study showed that CPG sites in monoamine oxidase A (MAOA) were significantly less methylated in PD patients than in healthy controls (13–15) and negative life events were associated with diminished levels of DNA methylation (14, 16, 17). Another study found that childhood abuse has a continuous effect on the changes in the methylation level of men and women (18–21). Moreover, Kang et al. noted that the methylation status of the SLC6A4 gene promoter region is significantly related to the severity of childhood abuse and clinical manifestations, indicating that the SLC6A4 methylation status may be a biological marker of childhood abuse and PD. The results of these previous studies indicate the importance of abnormal DNA methylation in the pathogenesis of PD, although the number of studies and the sample sizes have been limited, and most of the findings have not been confirmed in replication studies.

In a genome-wide association study (GWAS) conducted at the Max Planck Institute of Psychiatry in Munich, TMEM132D was first known as a probable candidate gene for PD. In a European GWAS, we found singlenucleotide polymorphisms (SNPs) in the TMEM132D relevant to PD (22). This result was supported by a replication study, which verified that TMEM132D is a susceptibility gene of PD (22, 23). A large-sample PD genome-wide association cohort study involving 1483 PD patients and 75379 normal controls found that PD may be associated with the rs1873727 polymorphism of TMEM132D intron 3 (22). This result duplicates the findings of a previous GWAS studies on PD. The function of TMEM132D gene has previously been analyzed in experiments involving rats with rich environments and chronic stress. In humans, experimenters have found that increased methylation of the promoter of TMEM132D gene may lead to decreased expression of single nucleotide polymorphisms (SNP) in the promoter region, thus leading to an increase in the sensitivity of individuals under the same environmental stress (15). The results of this study also indicated that the elevated methylation of the TMEM132D gene functional region may be potentially related to the occurrence and development of PD. The current research still does not clearly show how the types of childhood abuse are related to TMEM132D and panic disorder.

Childhood abuse, a public health problem that has attracted much attention at domestically and abroad, has been confirmed as a risk factor for the onset of PD. Childhood abuse is defined as various forms of adverse behavior committed by the guardian of a child, causing direct or potentially lasting significant harm to the child’s survival and development, including sexual abuse, physical abuse, emotional abuse, physical neglect and emotional neglect et al. (24). In China, the prevalence of childhood abuse is 30.5%, of which physical abuse accounts for 26.6%, sexual abuse accounts for 8.7%, emotional abuse accounts for 19.6%, and neglect accounts for 26.0% (25–27). Research by Lochner et al. showed that adults with a history of childhood abuse are more susceptible to anxiety disorders, depression, personality disorders, and other mental illnesses, suggesting that childhood abuse is a risk factor that increases the risk of panic disorder (28, 29). Previous studies have also reported the correlation between physical and sexual abuse and PD (30). One study administered the Childhood Trauma Questionnaire (CTQ) to investigate 90 PD patients who had experienced childhood abuse. They found that the detection rate of physical abuse was 40.7% and that of sexual abuse was 31.9% (31). The experience of physical abuse and sexual abuse not only increased the risk of PD, but it was also closely related to the severity of PD symptoms (32–34). The above studies all show that childhood physical abuse may have a certain predictive effect on PD. It is worth noting that there are also studies that hold different views. For example, a study from the Netherlands found that childhood abuse is not the cause of PD and has no significant effect on the persistence of PD (35).

In recent years, much attention has been paid to the study of the interaction between environment and heredity on the pathogenesis of diseases. A genome-wide association study (GWAS) found that 2,868 CpG loci were significantly altered in childhood maltreatment, which suggests the crucial role of epigenetic mechanisms in childhood maltreatment (36). A studie have demonstrated that childhood abuse plays a significant role in the pathophysiological mechanism of mental disorders through changes in the HPA axis (37). HPA axis reactivity may be more significant in PD patients with childhood maltreatment (38). The study found that DNA methylation in exon 1F promoter region of NR3C1 gene increased and NR3C1mRNA expression decreased in suicidal patients with a history of childhood abuse, and showed that the change of GR receptor expression was more closely related to childhood abuse than suicide factors (39). A study on the relationship between the methylation level of the promoter region of the serotonin transporter gene (SLC6A4) and childhood abuse found that childhood abuse had a sustained effect on methylation levels in both men and women (18). The methylation status of SLC6A4 gene promoter is significantly correlated with the severity of childhood abuse and clinical manifestations, indicating that SLC6A4 methylation status may be a biological marker of childhood abuse and PD (40). There are few domestic studies on the association between TMEM132D gene methylation and PD. Epigenetics, as a sign of the interaction between genes and environment, provides an opportunity to explore the molecular mechanism of childhood trauma on PD.

Therefore, this study explored the impact of TMEM132D gene methylation and childhood abuse on PD. Our hypotheses for this study were: childhood abuse experience is significantly associated with PD, TMEM132D gene methylation is significantly related to PD and TMEM132D gene methylation has a mediating effect between PD and childhood abuse experience. It is hoped that the results of this study will provide some evidence for understanding the pathogenesis of PD and also provide a certain theoretical basis for PD intervention.

## Material and methods

### Study design and samples

Case control group design was used in this study (Supplementary Table 1). PD patients were recruited from an outpatient unit at Nanjing Brain Hospital. PD was the main diagnosis, which was ascertained by trained psychiatrists according to the Diagnostic and Statistical Manual of Mental Disorders (DSM-IV) criteria. All patients were between the ages of 18 and 55, had primary school education or above and were right-handed. The PD patients had not taken any psychotropic medications for at least 6 months before admission, and scored more than 14 points in the Hamilton Anxiety Scale (HAMA). The exclusion criteria were as follows: patients with nervous system diseases, mental diseases or serious physical diseases; pregnant or lactating women; medical treatment such as medications, therapy, psychotherapy, electroconvulsive therapy and other physical therapy 6 months before entering the group; major life changes within the past year.

Control subjects were recruited from society. All subjects were between the ages of 18 and 55, had primary school education or above, were right-handed and had HAMA scores below 7 points. The exclusion criteria were as follows: patients with nervous system diseases, mental diseases or severe physical diseases; pregnant or lactating women; medical treatment such as medications, therapy, psychological counseling, electroconvulsive therapy or other physical therapy 6 months before entering the group; significant life changes within the past year. All subjects gave written informed consent.

### Questionnaires

*Sociodemographic and clinical characteristics* were assessed using the self-designed general data questionnaire, including name, gender, height, weight, age, place of birth, nationality, marital status, educational level, and monthly income per family.

*The Hamilton Anxiety Scale (HAMA)* was used to assess severity of anxiety. The HAMA is a 14-item evaluation measure in which adopt a 5-level soring method of 0–4 points. The standards of each level are none, mild, moderate, severe and extremely severe.

*Panic Disorder Severity Scale (PDSS)* was used to assess the severity of panic. The PDSS has 7-items, adopting a 5-level scoring method of 0–4 points. The standards of each grade are none, mild, moderate, severe and extreme, and the sum of the scores of each item is the total score of the scale. 8–10 is mild, 11–13 is moderate, 14–16 is heavy, more than 17 is severe.

*Childhood Trauma Questionnaire (CTQ)* was used to investigate Childhood trauma experience. The CTQ consists of 28 items, including 25 clinical items and 3 validity items. Five types of childhood abuse were evaluated, including emotional abuse, physical abuse, sexual abuse, emotional neglect and physical neglect. Each item adopts a five-level score, which reflects the frequency of abuse experience (never, occasionally, sometimes, often, always). Each abuse subscale scores 5–25 points, with a total score of 25–125 points. The higher the score of the questionnaire, the more child abuse experiences.

### Blood sample collection and methylation determination

#### DNA extraction and processing

5ml peripheral venous blood was collected from all subjects and plasma was separated after EDTA anticoagulation (Supplementary Table 2). The DNA was extracted using a desktop high-speed centrifuge (Eppendorf, Centrifuge 5417) and obtained through five steps: equilibrium adsorption column, sample processing, adsorption, rinsing, and elution. Store in a refrigerator at 4°C. The quantitative methylation analysis performed in this study used bisulfite amplicon sequencing (BSAS). DNA samples were first bisulfite-converted using a kit for the bisulfite conversion of DNA (EZ DNA Methylation Kit, Zymo Research, Irvine, CA, United States). Multiplex PCR was performed with optimized primer sets combination. The cycling temperature program was 98°C for 10 min, 64°C for 2.5 h. Mix 150 ul sample with 600 ul M-Binding Buffer. Then, the water was removed by centrifugation at full speed (13,000 rpm) for 30 se and the filtrate was removed by centrifugation at full speed (13,000 rpm) for 200 ulm-washbuffer. 200 ulm-desulfurization buffer was added and centrifuged at full speed (13000 rpm) for 30 seconds. The m-elution buffer was heated in a 60°C water bath, 200ulm m-elution buffer was added to the column, centrifuged at full speed (1300rpm) for 30 seconds, and the process was repeated. Then cover and let stand for 10–15 min. Finally, the m-elution buffer was removed, and 30ulm m-elution buffer was added to the column, and the DNA was eluted by centrifugation at full speed (13000 rpm).

#### PCR amplifications and MethylTarget methylation sequencing

Primers were designed with PyroMark Assay Design software. The required amount of converted DNA samples, specific positive and negative primers of amplified gene sequence and Pyromark PCR reagent kit were prepared into a suitable reaction system for PCR amplification. PCR amplicons were amplified and constructed for each target CPG region. The product was sequenced by illumina2 × 150 bp on the illumine Mi Seq table sequencer. The average sequencing depth of all amplifiers in 90% of the samples was more than 500 × c. The CPG test sites were named for their relative distance from the transcriptional initiation site (in bp). The ratio of methylated cytosine and total tested cytosine was calculated as the methylation level of each CPG site. “Target region sequencing depth” was the number of sequences/amplifiers compared to the target area. MethylTarget evaluation criteria: search for CPG islands (including all splicing modes) in the region 2K upstream of the gene transcription initiation site to 1K downstream of the first exon.

### Statistical analysis

The differences of continuous variables between groups were compared by independent sample *T*-test, and the general demographic data were compared by *Z*-test or χ-test. SPSS23.0 software was used to analyze the age, education level, HAMA score, PDSS score, CTQ score and its subscale score (significant level *p* < 0.05). There were no significant differences in some demographic information. The relationship between methylation of CpG site of TMEM132D gene, childhood trauma experience and severity of clinical symptoms in PD patients was investigated by Spearman correlation analysis and mediation model analysis (Supplementary Figure 1). The mediation model was analyzed by the PROCESS plug-in based on SPSS23.0 software. A 95% confidence interval (CI) without 0 indicates a significant mediation effect.

## Results

### Demographic information and clinical assessments

The analyzed sample comprised 32 PD patients and 22 healthy controls (HCs). Table 1 shows information on the general demographic data of PD group (16 males; age = 33.1 ± 7.4 years [mean ± SD]) and HCs group (age ± SD = 33.3 ± 7.2, 12 males; age = 33.3 ± 7.2 years [mean ± SD]). There were no significant differences in age, gender or education between the subjects with PD and HC (*p* > 0.05). There was also a significant difference in HAMA scores (*t* = −11.8, *p* < 0.001). Seven patients with PD did not fully complete the PDSS or clinical measures, thus the numbers included in further analysis varied in different categories. The mean PDSS score in patients was 14.8 (*n* = 25, SD = 5.7), ranging from 8 to 22. DNA methylation data were available for all 54 participants.

**TABLE 1:** Comparison of the demographic and clinical variables between panic disorder (PD) patients and healthy controls (HCs).

Characteristics	PD patients	HCs
N	32	22
Sex		
Male	50% (16)	54.5% (12)
Female	50% (16)	45.5% (10)
Age(SD)	33.1 (7.4)	33.3 (7.2)
Education(SD)	13.9 (3.3)	15.6 (3.8)
HAMA-T(SD)	20.3 (7.0)	2.2 (1.9)
PDSS score(SD)	14.8 (3.8)	NA

χ^2^ and P-values were obtained with the chi-squared test. T and P-values were obtained with two-sample t-tests. SD, standard deviation; PDSS, Panic Disorder Severity Scale; HAMA-T, total score of the Hamilton Anxiety Scale.

### Childhood abuse status

**FIGURE 1:**
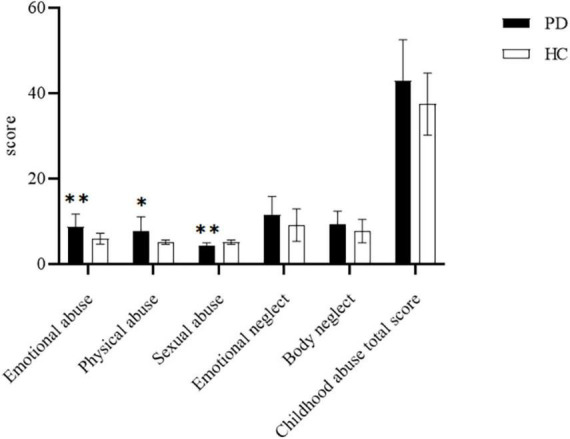
Comparison of scores of childhood abuse scale between the panic disorder (PD) group (*n* = 32) and healthy controls (HCs) group (*n* = 22).*P < 0.05;^**^P < 0.01.

We compared the childhood abuse with PD (*n* = 32) and HCs (*n* = 22) group using t-tests (Figure 1). As presented in Figure 1, there were significant differences in emotional abuse (*p* < 0.01), sexual abuse (*p* < 0.01), and physical abuse (*p* < 0.05) between the PD group and HCs group. Meanwhile, there was no significant difference in emotional neglect, body neglect and sexual abuse between PD and HCs groups (*p* > 0.05).

### The relationship between transmembrane protein 132D (TMEM 132D) gene CPG site methylation and panic disorder

**FIGURE 2:**
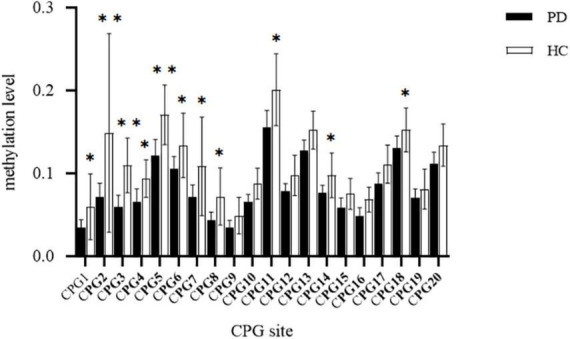
Comparison of methylation at CPG site of transmembrane protein132D (TMEM132D) gene in the panic disorder (PD) group and healthy control (HC) group, *p < 0.05;^**^p < 0.01.

Comparing the methylation of each CPG unit in the PD (*n* = 32) and HC (*n* = 22) groups by *t*-tests (Figure 2), we found that the PD group had significantly lower methylation at CpG1, CpG2,CpG3,CpG4,CpG5,CpG6,CpG7,CpG8,CpG11,CpG14 and CpG18 than did the HCs (*p* < 0.05). Hence, all subsequent epigenetic analysis were carried out only for these 11 CpG sites.

**FIGURE 3:**
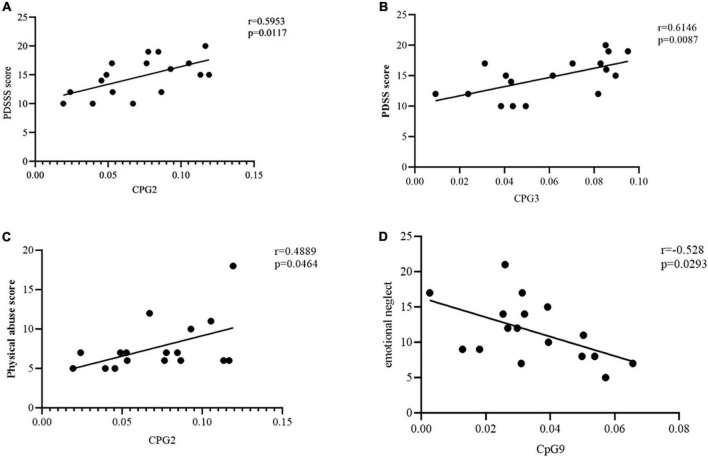
**(A)** Correlation between CpG2 site methylation of transmembrane protein132D (TMEM132D) gene and PDSS score. The threshold was set at a significance level of *p* < 0.05. PDSS, Panic Disorder Severity Scale. **(B)** Correlation between CpG3 site methylation of TMEM132D gene and PDSS score. The threshold was set at a significance level of *p* < 0.05. PDSS, Panic Disorder Severity Scale. **(C)** Correlation between CpG2 site methylation of TMEM132D gene and physical abuse score. The threshold was set at a significance level of *p* < 0.05. **(D)** Correlation between CpG9 site methylation of TMEM132D gene and emotional abuse score. The threshold was set at a significance level of *p* < 0.05.

We performed Pearson’s correlation analysis in PD group to explore the relationship between methylation at 11CpG sites and PDSS score. The CpG2 site in the priming region of TEME132D gene were positively associated with PDSS score (*r* = 0.5953, *p* = 0.0117, Figure 3A). The degree of CPG3 methylation was negatively correlated with PDSS score (*r* = 0.6146, *p* = 0.0087, Figure 3B). Other CpG sites were not associated with PDSS score.

We performed Pearson’s correlation analysis in PD group to verify the relationship between methylation at 11CpG sites and childhood abuse. The CpG2 site in the priming region of TEME132D gene were positively associated with physical abuse (*r* = 0.4889, *p* = 0.046, Figure 3C). The degree of CPG9 methylation was negatively correlated with emotional neglect (*r* = -0.528, *p* = 0.0293, Figure 3D). Other CpG sites were not associated with childhood abuse.

### The methylation of CPG site of transmembrane protein 132D (TMEM 132D) gene is the mediating effect of childhood abuse and panic symptoms

**FIGURE 4:**
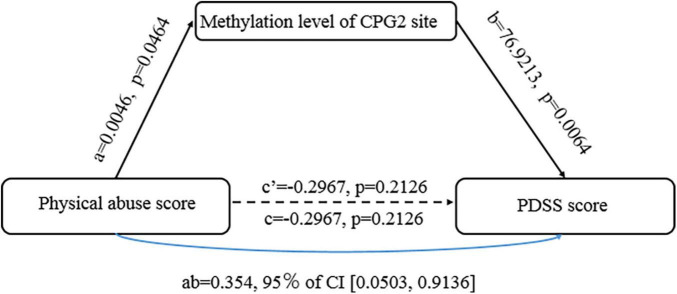
The mediating effect of transmembrane protein132D (TMEM132D) gene CPG site methylation in emotional abuse and panic symptoms.

Based on results above, we conducted an additional path analysis to examine whether a reduced TMEM132D methylation mediated the relationship between the physical abuse in childhood abuse and PD symptom severity. Figure 4 shows the mediation effect of the methylation of TMEM132D in the relationship between childhood abuse and PD. The mediation model was analyzed using the PROCESS plug-in based on SPSS 23.0 software. A 95% confidence interval (CI) without 0 indicates a significant mediation effect.

As shown in Figure 4, the methylation of CpG2 site of TMEM132D mediated the correlation between physical abuse and PD (*b* = 76.9213, [95%bootstrap CI 0.0503, 0.9136]). The results of the Bootstrap mediating procedure showed that the total effect of physical abuse on PD was 0.059 (Table 2). Under the mediating effect of CpG2, the direct effect of childhood physical abuse on PD score was -0.297, which was not statistically significant (*t* = −1.301, *P* > 0.05), thus CpG2 methylation play a fully mediating role in the relationship between physical abuse and PD symptoms.

**TABLE 2:** The mediating effect of transmembrane protein132D (TMEM132D) gene CPG site methylation in emotional abuse and panic symptoms.

	Effect size	Boot SE	Boot 95% CI
Total effect size	0.059	0.2518	[−0.4784, 0.5957]
Direct effect size	−0.297	0.2272	[−0.7841, 0.1906]
Indirect effect size	0.354	0.2179	[0.0245, 0.9313]

## Discussion

In this study, we reported that childhood abuse experiences, especially physical abuse, are significantly related to PD. We also found that the PD group showed lower TMEM132D CpG site methylation than did the HC group, which led us to examine the link between CPG site methylation and PD symptoms. The results showed that TMEM132D methylation at CPG2 and CpG9 had an apparent positive correlation with current PD symptoms. At the same time, TMEM132D methylation at CpG2 has a fully mediating effect between panic disorder and physical abuse.

We found that lower methylation level of the CpG island site of TMEM132D is significantly related to panic disorder, and thus may increase the risk of panic disorder. T-test results of the determination of methylation at CpG sites of TEME132D showed that the methylation levels of CpG1, CpG2, CpG3, CpG4, CpG5, CpG6, CpG7, CpG8, CpG11, CpG14, and CpG18 in the promoter region of TMEM132D were significantly lower in the panic disorder group than in the healthy control group. Correlation analysis of TEME132D CpG site methylation level with panic disorder suggested that TEME132D methylation in CpG2 may be a predictor of PD. This is consistent with the results of a large-sample panic disorder genome-wide association cohort study. The study involved a customized array of SNPs from 1483 panic disorder patients and 75379 normal controls. The study found that the rs1873727 polymorphism in intron 3 of the TMEM132D may be associated with PD (41). Their experiment repeated the findings of a previous PD GWAS study, and some preclinical trials and clinical sample studies were further repeated for verification. A rat experiment also reported the same conclusion. In that study, scholars analyzed the gene function of TMEM132D in a chronic stress and enriched environment. In several EWAS studies of panic disorder, it was found that 40 CpG loci were significantly associated with hypo-methylation, gender-specific methylation changes may exist in HECA gene, and the degree of cg19917903 methylation in CFAP46 gene decreased significantly (2). This conclusion suggests that hypo-methylation at the promoter region of TMEM132D may be a potential mechanism of panic disorder. Research suggests that TMEM132D is a candidate gene for PD onset, and the study of its methylation level can be used as a research direction to explore the physiological and pathological mechanisms of PD.

This study shows that childhood abuse experience, especially physical abuse, is significantly related to panic disorder. The comparison of childhood abuse between the PD group and the HC group showed that the two groups had significant differences in emotional abuse, physical abuse and sexual abuse, but no significant differences in emotional neglect body neglect and childhood abuse of total score. Thus, PD patients may have suffered more emotional abuse, physical abuse and sexual abuse during childhood. No differences from the control group were detected in emotional neglect and body neglect in this study.

Domestic studies have pointed out that PD patients have a variety of childhood traumatic experiences that are related to PD severity and impaired social function and sexual relations (26). However, physical and sexual abuse during childhood appear to be the clearest risk factors for developing PD (13, 42, 43). In our correlation analysis of childhood abuse and the severity of panic symptoms, we discovered the predictive effect of physical abuse on PD. This has shown consistent results with many studies. Bandelow and other studies have found that the occurrence of emotional abuse is often accompanied by a higher degree of physical abuse and emotional and physical neglect. Various forms of abuse may accompany emotional abuse, which can explain the high detection rate of emotional abuse (44, 45). There are many possible reasons for this contradictory result. It may be caused by insufficient sample size, or it may be related to differences in cultural regions. This reminds us that in the future it is necessary to take the local historical culture and social conditions into consideration to unify the definition and evaluation criteria of childhood trauma.

It is worth noting that the results of this study also indicate that TMEM132D methylation has a fully mediating effect between panic disorder and physical abuse. The interaction between TMEM132D methylation and physical abuse can predict panic disorder. When an individual suffers more physical abuse, the methylation level of CpG2 on TMEM132D may enhance expression, which affects the occurrence of panic disorder. This supports a study of epigenetic mechanisms of childhood trauma and disease risk. The study found through genome-wide methylation analysis that 2868CpG sites of childhood abuse have undergone meaningful changes (31). Research has proven that environmental factors and random variations *in vivo* can affect the epigenetic state of adult organisms. Many studies have reported the impact of the interaction between gene methylation and environmental factors on the disease (42, 43). Because the epigenetic modification state is heritable, it can directly affect the characteristics of gene transcription. These modified states can be inherited to progeny cells through mitosis, which has a long-term and stable impact on the disease phenotype and susceptibility (13, 46, 47).

In summary, childhood abuse is a risk factor for PD (20, 48), and abnormal expression of gene methylation increases the individual’s susceptibility in risk environments, thereby increasing the prevalence of PD. Therefore, society and caregivers should create a positive environment that is conducive to the growth of children. It is imperative to be aware of the impact of abuse (including emotional abuse, physical abuse, sexual abuse, emotional neglect, and physical neglect) on children and minimize factors in the environment that hinder children’s growth so that they can grow up healthy and happy (49–51). In clinical work, trauma assessment should be part of the clinical interview. Psychological counselors or therapists should take into account that PD patients may have experiences of childhood abuse, and should provide necessary psychological treatment and intervention.

This study has some limitations. Frist, the sample size of the study was small and as a result, statistical power may have been limited. In the future, a larger sample size is needed to verify the research results. Second, peripheral blood may not necessarily reflect the levels of DNA methylation in the CNS despite previous evidence suggesting that the DNA methylation patterns in peripheral blood cells and several brain areas are highly comparable (52, 53). Finally, in this study, a cross-sectional study was used. It was not clear from cross-sectional studies whether adverse environmental factors caused changes in biomarkers or whether genetic basis increased susceptibility to adverse environments.

## Conclusion

We performed multiple PCR amplification of the TMEM132D promoter fragment and identified that the methylation level of the CpG site in the TMEM132D promoter region was reduced in the PD group compared to the HC group. Our study also demonstrated that methylation level of the CpG2 site in the promoter region of the TMEM132D plays a fully mediating role in the process of physical abuse on PD symptoms, thus suggesting its further application in early intervention and treatment of panic disorder.

## Data availability statement

The original contributions presented in this study are included in the article/Supplementary material, further inquiries can be directed to the corresponding author.

## Ethics statement

The studies involving human participants were reviewed and approved by the Ethics Committee of the Nanjing Brian Hospital, affiliate of Nanjing Medical University. The patients/participants provided their written informed consent to participate in this study. Written informed consent was obtained from the individual(s) for the publication of any potentially identifiable images or data included in this article.

## Author contributions

QY made substantial contributions to initiate, and wrote the manuscript. As well as senior fellow apprentice YW who ever made a lot of contribution on collection of participants. YW made substantial contributions in recruiting participants. HX and HD made substantial contirbutions in collecting image data. QY revised it critically for important intellectual content with suggestions from all authors. ChuW led research training, and, along with NL and NZ, provided editing and writing assistance. QY agreed to be accountable for all aspects of the work in ensuring that questions related to the accuracy or integrity of any part of the work are appropriately investigated and resolved. All authors contributed to the article and approved the submitted version.
