# Supplementary material for: The mediating role of transmembrane protein 132D methylation in predicting the occurrence of panic disorder in physical abuse
Source: Front Psychiatry. 2022 Aug 11;13:972522. doi: 10.3389/fpsyt.2022.972522 (PMC9403743; doi:10.3389/fpsyt.2022.972522)
Supplement: Supplementary file 1 [file Data_Sheet_1.docx]

**Table2.1** Main reagents needed for experiment

| Product name | Item No. | Producer |
| --- | --- | --- |
| PCR Accessory Set | 11327 | Sequenom |
| Iplex Gold Reagent kit（Large） | 10136 | Sequenom |
| SpectroCHIP II&Resin kit（10*384 chip） | 10117-2 | Sequenom |
| Primer |  | Shanghai tianhao biology |

**Table2.2** Main instrument consumables

| Instrument name | Source of instrument | Model |
| --- | --- | --- |
| Table high-speed centrifuge | Eppendorf | Centrifuge 5417 |
| PCR instrument | MJ research | PTC200 |
| Pipette | Gilson | 1000μL、200μL、10μL |
| Time of flight mass spectrometer | Sequenom | Mass MARRAY |
| Sample spotting instrument | Sequenom | Mass MARRAY |
| Various models of tip, PCR tube and centrifuge tube | Axygen |  |


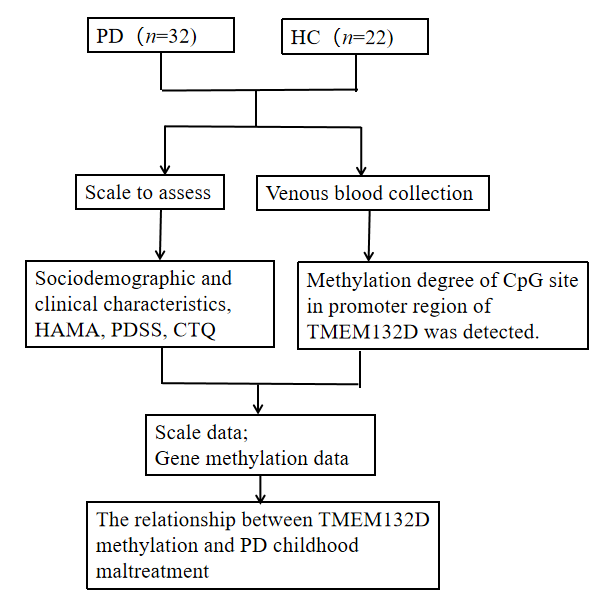


Supplement figure.1 experimental process diagram.
